# Changes in Waist Circumference and the Incidence of Acute Myocardial Infarction in Middle-Aged Men and Women

**DOI:** 10.1371/journal.pone.0026849

**Published:** 2011-10-26

**Authors:** Tina Landsvig Berentzen, Marianne Uhre Jakobsen, Jakob Gerhard Stegger, Jytte Halkjaer, Anne Tjønneland, Thorkild I. A. Sørensen, Kim Overvad

**Affiliations:** 1 Institute of Preventive Medicine, Copenhagen University Hospital, Copenhagen, Denmark; 2 Department of Epidemiology, School of Public Health, Aarhus University Aarhus, Denmark; 3 Department of Cardiology, Center for Cardiovascular Research, Aalborg Hospital, Aarhus University Hospital, Aalborg, Denmark; 4 The Danish Cancer Society, Institute of Cancer Epidemiology, Copenhagen, Denmark; Lerner Research Institute, Cleveland Clinic, United States of America

## Abstract

**Background:**

Waist circumference (WC) measured at one point in time is positively associated with the risk of acute myocardial infarction (MI), but the association with changes in WC (DWC) is not clear. We investigated the association between DWC and the risk of MI in middle-aged men and women, and evaluated the influence from concurrent changes in BMI (DBMI).

**Methodology/Principal Findings:**

Data on 38,593 participants from the Danish Diet, Cancer and Health study was analysed. Anthropometry was assessed in 1993–97 and 1999–02. Information on fatal and non-fatal MI was obtained from National Registers. Cases were validated by review of the medical records. Hazard ratios (HR) were calculated from Cox proportional hazard models with individuals considered at risk from 1999–02 until December 30 2009. During 8.4 years of follow-up, 1,041 incident cases of MI occurred. WC was positively associated with the risk of MI, but weakly after adjustment for BMI. DWC was not associated with the risk of MI (HR per 5 cm change  = 1.01 (0.95, 1.09) with adjustment for covariates, baseline WC, BMI and DBMI). Associations with DWC were not notably different in sub-groups stratified according to baseline WC or DBMI, or when individuals with MI occurring within the first years of follow-up were excluded.

**Conclusions/Significance:**

WC was positively associated with the risk of MI in middle-aged men and women, but changes in WC were not. These findings suggest that a reduction in WC may be an insufficient target for prevention of MI in middle-aged men and women.

## Introduction

Obesity and weight gain are strong risk factors for coronary heart disease (CHD) [1]. Weight loss improves the cardiovascular risk factor profile [2]; [3], but most long-term population based studies suggest an increased risk of CHD with weight loss [4]–[8]. Pre-existing or sub-clinical diseases and high-risk behaviors (as smoking) have been suggested to explain the increased risk of CHD associated with weight loss, but the risk persist after careful adjustment for these factors [4]–[8].

Individuals differ in their regional distribution of body fat, which have implications for their morbidity and mortality. Anthropometric measures of abdominal fatness (e.g. waist circumference (WC)) appear to be more strongly associated with the risk of CHD than anthropometric measures of general fatness (e.g. body mass index (BMI)) [9]–[20]. This has predominantly been attributed to accumulation of intra-abdominal fat, which is strongly associated with cardiovascular risk factors and possibly also with incident CHD [21]–[23]. In contrast, anthropometric measures of peripheral fatness are inversely associated with the risk of CHD [24] possibly due to cardio-protective effects of the skeletal muscle and the gluteofemoral fat [25]. Furthermore, two recent studies found that weight loss was associated with increased all-cause and CHD mortality [11]; [26], whereas waist loss was associated with decreased mortality indicating that loss of abdominal fat mass with preservation of other body compartments is beneficial.

Although several studies have shown that WC measured at one point in time is associated with the risk of CHD, it is unclear whether changes in WC (DWC) are associated with the risk of CHD. We therefore investigated the association between DWC and the risk of acute myocardial infarction (MI) in a large cohort of middle-aged men and women, and evaluated the influence from concurrent changes in BMI (DBMI).

## Methods

In 1993–97, 160,725 individuals aged 50–64 years with no previous cancer diagnosis were invited into the Danish prospective study ‘Diet, Cancer and Health’ (figure S1). A total of 57,053 participants accepted the invitation. Participants filled in questionnaires and were clinically examined (569 were later excluded due to a cancer diagnosis, which was not, due to processing delays, registered at the time of the invitation). In 1999–2002, repeated information was collected by questionnaires. Details of the study were described previously [27]; [28].

### Ethics statement

The Danish Data protection Agency and the regional Ethical Committee approved the study, which was in accordance with the Helsinki Declaration II. Participants signed a written consent before participating.

### Outcome

Cases of nonfatal and fatal MI (International Classification of Disease (ICD) 8: 410–410.99 or ICD10: I21.0–I21.9) were identified by linkage with the Danish National Patient Register [29] and the Danish Causes of Death Register [30] via the unique identification number assigned to all Danish citizens. Sudden cardiac death (ICD8: 427.27 or ICD10: I46.0–I46.9) was also included if the cardiac arrest was believed to have been caused by MI. From the date of enrolment into the cohort and until December 31st 2003, cases were validated by review of medical records in accordance with the guidelines of the American Heart Association and the European Society of Cardiology [31]. From January 1st 2004 and until December 30 2009, and for participants whose medical records had not been available in the previous period, participants with a diagnosis of MI from a hospital ward were accepted as cases without validation, as this diagnosis had a positive predictive value above 90% in the Patient Register [31]. All other cases were individually validated by review of diagnoses and procedure codes in the Patient Register and the Causes of Death Register.

### Exposure

In 1993–97, technicians measured the individuals' height (nearest 0.5 cm without shoes) and weight (nearest 0.1 kg using a digital scale, with light clothes/underwear). The WC was measured (nearest 0.5 cm) with a measuring tape at the smallest horizontal circumference between the ribs and the iliac crest (natural waist), or, in case of an indeterminable WC narrowing, halfway between the lower rib and the iliac crest. In 1999–02, individuals received a self-administrated questionnaire and reported their weight (kg) and WC (cm) measured at the level of the umbilicus using an enclosed paper measuring tape. BMI (kg/m2) was calculated as weight per height squared. Change in BMI (DBMI) (kg/m2) and change in WC (DWC) (cm) was calculated as the value in 1993–97 subtracted from the value in 1999–02.

### Covariates

Covariates, assessed with the 1999–02 questionnaire, were included in the analyses: *smoking* (never, ex, current smoker of <15 g/day, 15–25 g/day, >25 g/day), *sports activity* (0, >0 hours/wk) [32]; [33], *total energy intake (including alcohol)* (KJ/day) [32]; [34], *Mediterranean diet score* [32]; [34]; [35], *drinking pattern* (abstainer, 0–3 times/month, 1–4 times/wk, 5–6 times/wk, daily) and *length of education* (<8 years, 8–10 years, >10 years) [27].

Chronic disease may induce changes in anthropometry and increase the risk of MI [4]–[8]. Chronic disease *(yes/no)* occurring before examination in 1999–02 was therefore included as a covariate. Chronic disease was defined according to a selection of ICD8 and ICD10 codes representing chronic somatic disease [36]. Information on diagnosed diseases was obtained by linkage to the National Patient Register [29] and the National Diabetes Register [37].

### Exclusions

Individuals for whom questionnaires were incomplete were excluded, and so were individuals with a diagnosis of MI before examination in 1999–02 (figure S1).

Misreporting may be most pronounced in individuals with extreme measurements so individuals with values below the 0.5 and above the 99.5 sex-specific percentiles of BMI and WC, and below the 2.5 and above the 97.5 sex-specific percentiles DBMI and DWC were excluded (figure S1). These cut-off values were chosen to reduce the influence of outliers on the associations.

### Statistical Analyses

Analyses were conducted in STATA version 9.2 (Stata Corporation, College Station, Texas; www.stata.com).

Hazard ratios (HR) and 95% confidence intervals of MI were calculated from Cox's proportional hazard models. Years since examination in 1999–02 were used as the time axis. Thus, individuals were considered at risk from 1999–02 until time at MI, death from other causes, emigration/disappearance or December 30 2009, whichever came first. Analyses were conducted for each sex separately, and combined if appropriate. Sex differences were formally tested on the multiplicative scale by cross-product terms using Wald tests.

BMI in 1993–97 was included as restricted cubic splines (3 knots) [38]; [39] in models with age in 1999–02, years between examinations and chronic disease. Covariates were added in a second step and WC in 1993–97 was added in a third step. Similar analyses were conducted for WC in 1993–97 with BMI added in the third step, and for BMI and WC measured in 1999–02. The DBMI was included as restricted cubic splines (3 knots) in models with age in 1999–02, years between examinations, BMI in 1993–97 and chronic disease. Covariates were added in a second step and DWC and WC in 1993–97 were added in a third step. Similar analyses were conducted for DWC with BMI and DBMI added in the third step. Splines were plotted to visually assess the shape of the associations, and associations were formally tested by Wald tests. Continuous covariates were also included as restricted cubic splines (3 knots). BMI, WC, DBMI and DWC were also examined as continuous linear variables in models with covariates added in the three steps described above. The proportional hazard assumption was assessed with a test based on Schoenfeld residuals, and no appreciable violations of the assumption were detected.

### Subgroups analyses

To explore if the association between DWC and MI was consistent throughout the range of the DBMI, associations between DWC and MI were investigated in groups with loss (DBMI< = 0) and gain in BMI (DBMI>0). Similarly, the association between DWC and MI was investigated in groups with a high and low WC in 1993–97 (cut-off at the sex-specific median of WC).

Atherosclerosis may go undiagnosed for years [40]; [41] and induce changes in anthropometry. This implies risks of bias due to reverse causality, which we explored by exclusion of cases occurring in the first one to five years of follow-up.

## Results

Between the examinations in 1993–97 and 1999–02, 1,778 individuals died and 460 emigrated/disappeared leaving 54,246 eligible for re-invitation. Among these, 5,865 did not respond, 2,858 did not want to participate and for 1,699 we had incomplete information on anthropometry and covariates. Moreover, 1,006 were excluded due to MI occurring before examination in 1999–02, and 4,225 were excluded due to extreme values on the anthropometric variables. Thus, 17,964 men and 20,629 women were eligible for the current study (figure S1).

In 1993–97, the median WC was 95 cm in men and 79 cm in women (table 1). During the 5 years between the examinations, the increase in WC was 3 cm in men and 7 cm in women (table 1). In men, 5,774 (32%) had a loss in WC and 12,190 (68%) had a gain in WC. In women, 3,268 (16%) had a loss in WC and 17,361 (84%) had a gain in WC. The Pearson correlation between BMI and WC was 0.85 in both sexes, and 0.44 and 0.37 between DBMI and DWC in men and women, respectively.

**Table 1:** Characteristics of the participants.

	Men (n = 17,964)	Women (n = 20,629)
	Median (5–95%tile)	Median (5–95%tile)
Age (year) in 1993–97	55.9 (50.7∶64.1)	56.1 (50.8∶64.2)
Age (year) in 1999–02	61.3 (56.0∶69.5)	61.5 (56.0∶69.5)
Years btw examinations in 1993–97 and 1999–02	5.3 (5.0∶5.9)	5.3 (5.0∶5.9)
Years btw examination in 1999–02 and end of follow-up	8.3 (4.0∶9.4)	8.4 (6.8∶9.5)
BMI (kg/m^2^) in 1993–97	25.9 (21.7∶31.9)	24.5 (20.1∶32.4)
BMI (kg/m^2^) in 1999–02	25.9 (21.6∶31.9)	24.5 (19.9∶32.4)
WC (cm) in 1993–97	94 (82∶110)	79 (67∶100)
WC (cm) in 1999–02	97 (85∶113)	87 (72∶109)
Changes in BMI (kg/m^2^) btw 1993–97 and 1999–02	0.0 (−1.9∶1.8)	−0.1 (−2.3∶2.1)
Changes in WC (cm) btw 1993–97 and 1999–02	3 (−6∶11)	7 (−3∶19)
Mediterranean Diet Score in 1999–02	5 (3, 7)	5 (3, 7)
Energy Intake (Mj/d) in 1999–02	10.2 (6.5, 15.3)	8.1 (5.2, 12.5)
Chronic diseased in 1999–02	29%	31%
Current smokers in 1999–02	30%	25%
Physically inactive in 1999–02	43%	37%
Daily alcohol intake in 1999–02	33%	19%
Less than 8 years of school in 1993–97	32%	29%

Abbreviations: BMI, body mass index. WC, waist circumference.

During a median follow-up of about 8 years, 739 new cases of MI occurred among men and 305 occurred among women.

### Single measurements of BMI and WC

The association between BMI in 1993–97 and MI was positive in both sexes, but weak after adjustment for WC. For the sexes combined, the HR per one kg/m^2^ was 1.03 (1.00, 1.07) after adjusting for covariates and WC (table 2, figure S2). The association between WC in 1993–97 and MI was positive in both sexes, but the association was weak after adjustment for BMI. For the sexes combined, the HR per 5 cm WC was 1.03 (0.97, 1.10) after adjusting for covariates and BMI (table 2, figure S2). Similar results were found for BMI and WC measured in 1999–02 (table 2, figure S3). None of the associations were notably different between men and women (interaction, *P>*0.5).

**Table 2:** Hazard ratios (HR) and 95% confidence intervals (CI) of myocardial infarction according to body mass index (BMI) and waist circumference (WC).

	Crude	Adjusted	Adjusted + WC	Adjusted + BMI
1993–97	HR (95%CI)*	HR (95%CI)*,†	HR (95%CI)*,†, ‡	HR (95%CI)*,†, ‡
BMI (kg/m2) in all participants	1.05 (1.03, 1.07)	1.04 (1.03, 1.07)	1.03 (1.00, 1.07) §	
BMI (kg/m2) in men	1.06 (1.04, 1.09)	1.06 (1.03, 1.09)	1.05 (1.01, 1.10)	
BMI (kg/m2) in women	1.03 (1.00, 1.06)∥	1.03 (1.00, 1.06)∥	1.00 (0.94, 1.05)	
WC (5 cm) in all participants	1.10 (1.06, 1.13)	1.09 (1.05, 1.12)		1.03 (0.97, 1.10)§
WC (5 cm) in men	1.11 (1.07, 1.16)	1.10 (1.06, 1.15)		1.02 (0.94, 1.10)
WC (5 cm) in women	1.07 (1.01, 1.13)∥	1.06 (1.01, 1.12)∥		1.06 (0.95, 1.17) ∥

1999–02				
BMI (kg/m2) in all participants	1.04 (1.02, 1.06)	1.04 (1.02, 1.06)	1.03 (1.00, 1.06) §	
BMI (kg/m2) in men	1.05 (1.03, 1.08)	1.05 (1.02, 1.08)	1.05 (1.01, 1.08)	
BMI (kg/m2) in women	1.03 (1.00, 1.06)∥	1.02 (0.99, 1.06)∥	1.01 (0.97, 1.06)∥	
WC (5 cm) in all participants	1.07 (1.04, 1.11)	1.07 (1.03, 1.10)		1.03 (0.98, 1.09) §
WC (5 cm) in men	1.08 (1.03, 1.13)	1.07 (1.03, 1.12)		1.01 (0.98, 1.08)
WC (5 cm) in women	1.07 (1.01, 1.12)	1.06 (1.01, 1.12)		1.07 (0.99, 1.17)

Abbreviations: BMI, body mass index. CI, confidence interval. HR, hazard ratio. WC, waist circumference.

*Adjusted for years between examinations, age, chronic diseases, sex (combined analyses).

† Adjusted for smoking, Mediterranean diet score, energy intake, education, drinking pattern, sports activity.

‡ WC added to analyses of BMI. BMI added to analyses of WC.

§ Associations were not notably different in men and women.

∥ Associations were accepted to be linear, except ^∥^.

### Changes in BMI and WC

**Figure 1:**
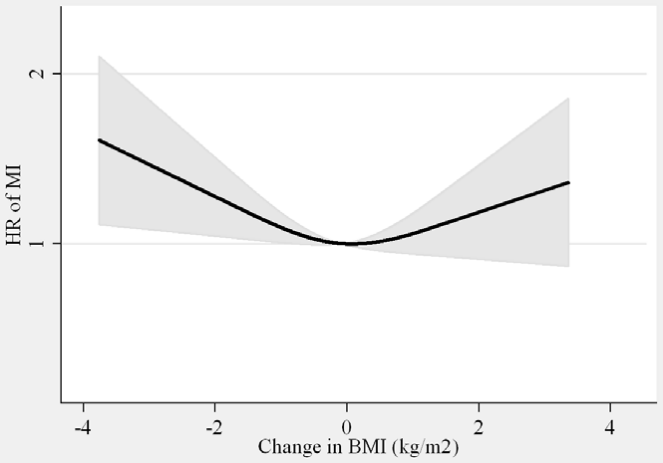
Hazard ratios (HR) and 95% confidence intervals (CI) of myocardial infarction (MI) according to changes in body mass index (BMI). Abbreviations: BMI, body mass index. HR, hazard ratio. MI, myocardial infarction Lines are the hazard ratios (shaded areas the 95% confidence intervals) derived from Cox proportional hazard models with changes in changes in body mass index included as restricted cubic splines (3 knots). Reference points are the mean of changes in body mass index. Adjusted for: sex, years between examinations, age, chronic diseases, smoking, Mediterranean diet score, energy intake, education, drinking pattern, sports activity, body mass index in 1993–97, waist circumference in 1993–97 and changes in waist circumference.

**Figure 2:**
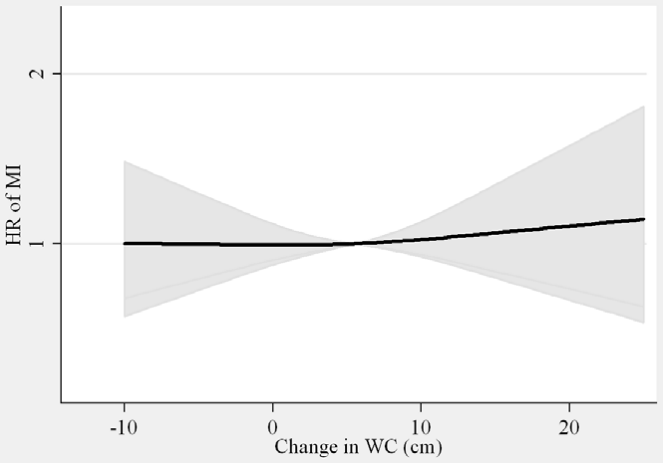
Hazard ratios (HR) and 95% confidence intervals (CI) of myocardial infarction (MI) according to changes in waist circumference (WC). Abbreviations: HR, hazard ratio. MI, myocardial infarction. WC, waist circumference Lines are the hazard ratios (shaded areas the 95% confidence intervals) derived from Cox proportional hazard models with changes in waist circumference included as restricted cubic splines (3 knots). Reference points are the mean of changes in waist circumference. Adjusted for: sex, years between examinations, age, chronic diseases, smoking, Mediterranean diet score, energy intake, education, drinking pattern, sports activity, body mass index in 1993–97, waist circumference in 1993–97 and changes in body mass index.

The association between DBMI and MI was U-shaped with the nadir of the curve at DBMI  = 0 (figure 1). Thus, for those with loss of BMI (DBMI< = 0) one kg/m^2^ decrease in BMI was associated with an 11% (HR = 1.11 (1.02∶1.22)) higher risk of MI, whereas for those with gain in BMI (DBMI>0) one kg/m^2^ increase in BMI was associated with an 8% (HR = 1.08 (0.97∶1.19) higher risk of MI with adjustment for covariates, DWC, BMI and WC in 1993–97. The DWC was not associated with MI (figure 2, table 3). Among all participants, the HR per 5 cm change was 1.00 (0.94, 1.07) with adjustment for covariates and WC in 1993–97, and 1.01 (0.95, 1.09) with additional adjustment for BMI in 1993–97 and DBMI (table 3). None of the associations were notably different between men and women (interaction, *P>*0.5).

**Table 3:** Hazard ratios (HR) and 95% confidence intervals (CI) of myocardial infarction according changes in waist circumference (DWC).

	Crude	Adjusted	Adjusted + DBMI
	HR (95%CI)*	HR (95%CI)*, †	HR (95%CI)*, †, ‡
DWC (5 cm) in all participants	1.00 (0.95, 1.06)	1.00 (0.94, 1.07)	1.01 (0.95, 1.09) §
DWC (5 cm) in men	1.00 (0.93, 1.08)	0.99 (0.91, 1.07)	1.02 (0.94, 1.11)
DWC (5 cm) in women	1.02 (0.94, 1.10)	1.02 (0.93, 1.12)	1.01 (0.92, 1.11)

Abbreviations: CI, confidence interval. DBMI, changes in body mass index. DWC, changes in waist circumference HR, hazard ratio.

*Adjusted for years between examinations, age, chronic diseases, waist circumference in 1993–97, sex (combined analyses).

† Adjusted for smoking, Mediterranean diet score, energy intake, education, drinking pattern, sports activity.

‡ Adjusted for changes in body mass index and body mass index in 1993–97.

§ Associations were not notably different in men and women.

Associations were accepted to be linear.

### Subgroups analyses

The DWC was not consistently associated with MI in the two strata of DBMI. The HR was 1.03 (0.95, 1.12) per 5 cm in participants with concurrent loss of BMI and 0.99 (0.91, 1.09) per 5 cm in participants with concurrent gain in BMI with adjustment for covariates, DBMI, BMI and WC in 1993–97.

The DWC was neither consistently associated with MI in the two strata of WC in 1993–97. The HR per 5 cm was 1.05 (0.95, 1.16) in participants with low WC and 1.00 (0.93, 1.08) in participants with high WC with adjustment for covariates, DBMI, BMI and WC in 1993–97.

Exclusion of cases of MI occurring within the first to five years of follow-up had no notable influence on the associations between DWC and MI (table S1).

## Discussion

This prospective study of middle-aged men and women showed that WC was positively associated with the risk of MI, but the association was weak after adjustment for BMI. DWC were not associated with the risk of MI, and this association was not altered by adjustment for covariates and DBMI, nor in groups defined according to WC in 1993–97, loss and gain in BMI, or when cases of MI occurring within the first one to five years of follow-up were excluded.

The strengths of the study are the large, well-characterized study population with anthropometry assessed at two time points. Selection bias is unlikely to have affected the results, as all study participants were followed after their second measurement of anthropometry until death or end of follow-up, and the number of participants lost due to death was low [29]; [30]. Cases of MI were validated by review of the medical records independently of the collection of anthropometry [31] whereby the risk of information bias is low.

Atherosclerosis may go undiagnosed for years [40]; [41] and its presence may induce changes in anthropometry. This implies risks of bias due to reverse causality, which we aimed to eliminate with our prospective design. We also conducted analyses where cases of MI in the first one to five years of follow-up were excluded. The exclusions had no notable influence on the associations, but we may have had insufficiently long follow-up and too few cases to fully address this. Other diseases may also both induce changes in anthropometry and affect the risk of MI [4]–[8]. We adjusted for chronic diseases [36] diagnosed before follow-up examination in 1999–02, but this had no notable influence on the estimates. The registries used to identify these diseased individuals cover our entire cohort and are valid [29]. Individuals with sub-clinical or psychiatric diseases are, however, not identified. We can therefore not fully exclude the influence from such diseases, but find it unlikely that several diseased individuals would participate in a long-term cohort study, which is supported by the low morbidity and mortality in the cohort [27].

Covariates that could have confounding or modifying effects were also included in the study, but these had no notable effects on the associations. Residual confounding from these, or confounding from other risk factors cannot be excluded, but the detailed data available makes it unlikely.

Trained technicians measured anthropometry in 1993–97, and measurement problems may have minimal impact on these results. Anthropometry was self-reported in 1999–02, but strong, quantitatively consistent associations between MI and both 1993–97 and 1999–02 measures were observed. This shows that the self-measured data, reported in a questionnaire, are as valid as the examiner-measured data in terms of predicting risk of MI. The use of different methods may, however, still have implications for the analysis and interpretation of changes in WC. A validation study within the cohort [42] found that the mean change in WC was somewhat overestimated in women (2.1 cm) and underestimated in men (0.8 cm). The difference was associated with BMI and WC, but it was concluded that the two measures could be used together in analyses of DWC if the statistical models included BMI and WC [42]. Accordingly, we included BMI and WC in analyses of DWC. We also excluded individuals with extreme anthropometric measurements as misreporting may be most pronounced in these individuals. Perhaps more important, information on WC was collected years before information about MI. It is thus unlikely that the over/underestimation of DWC is directly related to MI, which limits the risk of bias. Still we cannot exclude that errors have attenuated the results. DWC was, however, positively associated with all-cause mortality in the cohort [26] and we therefore expect that the used measure of DWC would capture most of the effects on MI.

Fatness, and in particular abdominal fatness, is assumed to increase the risk of MI [9]–[22]. This was also shown in our study, as WC was positively associated with the risk of MI. The association was, however, weak after adjustment for BMI as also observed in some previous prospective studies [9]–[19]. Adjustment for BMI in analyses of WC may reduce confounding from overall fatness, but the adjustment does also introduce a substitution aspect in the interpretation of the results. The risk of MI associated with a high WC in the adjusted model may reflect the effects of high amounts of abdominal fat or low amounts of gluteofemoral fat or lean body mass. In this regard, it is noteworthy that anthropometric measures of peripheral fatness are inversely associated with the risk of CHD after adjustment for BMI [24]; [25].

Changes in WC were not associated with the risk of MI, and adjustment for DBMI had no notable influence on this association possibly due to the modest correlation between DWC and DBMI. Accordingly, our findings suggest that it is not possible to predict the risk of MI associated with changes in WC from the risk associated with differences in WC measured at one point in time. The association with WC at one point in time may reflect lifelong exposure, whereas the risk associated with changes in WC may reflect the individual possibility to modulate such lifelong risk during a short five-year period.

A previous study [11] found that DWC were positively associated with the risk of mortality from CHD in postmenopausal women with heart disease, but only among women assigned to hormone therapy and who were in the extreme five percent of the waist change distribution. Estimates adjusted for overall weight change were not shown. The association between DWC and CHD may depend on various factors such as sex, age and health status of the study individuals [4]–[9]. Our participants were 50–64 years at baseline. It may hence be suspected that they already had redistributed fat mass to the abdominal fat depots [43] and therefore were too old to influence their risk of MI by modest changes in WC. DWC may also have different impact on morbidity and mortality from CHD with stronger associations for mortality [17], as also indicated in our cohort where DWC were positively associated with all-cause mortality [26]. This could explain the differences between these [11] and our findings.

**In conclusion**, WC was positively associated with the risk of MI in middle-aged men and women, but the association was weak after adjustment for BMI. DWC was not associated with the risk of risk of MI, and this association was not notably affected by adjustment for changes in BMI. According to these findings it is not possible to predict the risk of MI following changes in WC from studies where WC is only measured at one point in time. A reduction in WC may hence be an insufficient target for prevention of MI in middle-aged men and women.

## Supporting Information

Figure S1 **The study population.**
(PDF)

Figure S2 **Hazard ratios (HR) and 95% confidence intervals (CI) of myocardial infarction (MI) according to body mass index (BMI) and waist circumference WC) in 1993–97 with mutual adjustment.** Abbreviations: BMI, body mass index. HR, hazard ratio. MI, myocardial infarction. WC, waist circumference. Lines are the hazard ratios (shaded areas the 95%-confidence intervals) derived from Cox proportional hazard models with BMI and WC included as restricted cubic splines (3 knots). Reference points are the means of BMI and WC. Adjusted for: sex, years between examination, age, chronic diseases, smoking, WC (only BMI) and BMI (only WC)
(PDF)

Figure S3 **Hazard ratios (HR) and 95% confidence intervals (CI) of myocardial infarction (MI) according to body mass index (BMI) and waist circumference WC) in 1999–02 with mutual adjustment.** Abbreviations: BMI, body mass index. HR, hazard ratio. MI, myocardial infarction. WC, waist circumference. Lines are the hazard ratios (shaded areas the 95%-confidence intervals) derived from Cox proportional hazard models with BMI and WC included as restricted cubic splines (3 knots). Reference points are the means of BMI and WC. Adjusted for: sex, years between examination, age, chronic diseases, smoking, WC (only BMI) and BMI (only WC).
(PDF)

Table S1 **Hazard ratios (HR) and 95% confidence intervals (CI) of myocardial infarction according to changes in waist circumference (DWC) when cases occurring in the first one to five years of follow-up are excluded.** Abbreviations: CI, confidence interval. DWC, changes in waist circumference. HR, hazard ratio. * Adjusted for sex, years between examinations, age, chronic diseases, body mass index and waist circumference in 1993–97, changes in body mass index, smoking, Mediterranean diet score, energy intake, education, drinking pattern, sports activity.
(PDF)
