# Supplementary figures and images for: Changes in Waist Circumference and the Incidence of Acute Myocardial Infarction in Middle-Aged Men and Women
Source: PLoS One. 2011 Oct 26;6(10):e26849. doi: 10.1371/journal.pone.0026849 (PMC3202570; doi:10.1371/journal.pone.0026849)

**Figure S1. The study population**

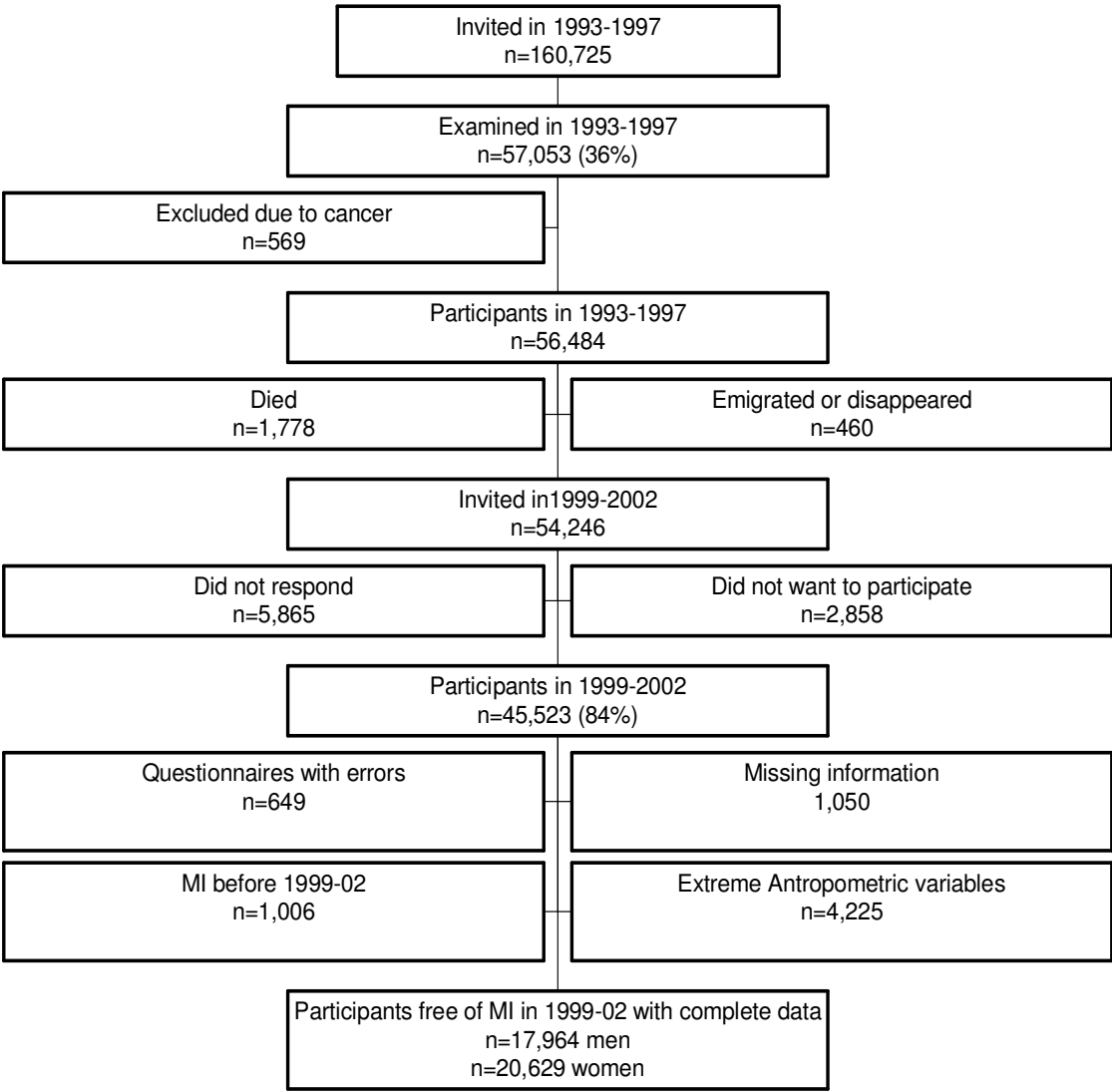

Supplement: Figure S1 — The study population. (PDF) [file pone.0026849.s001.pdf]
