# Supplementary material for: Changes in Waist Circumference and the Incidence of Acute Myocardial Infarction in Middle-Aged Men and Women
Source: PLoS One. 2011 Oct 26;6(10):e26849. doi: 10.1371/journal.pone.0026849 (PMC3202570; doi:10.1371/journal.pone.0026849)

Figure S3. Hazard ratios (HR) and 95% confidence intervals (CI) of myocardial infarction (MI) according to body mass index (BMI) and waist circumference WC) in 1999-02 with mutual adjustment

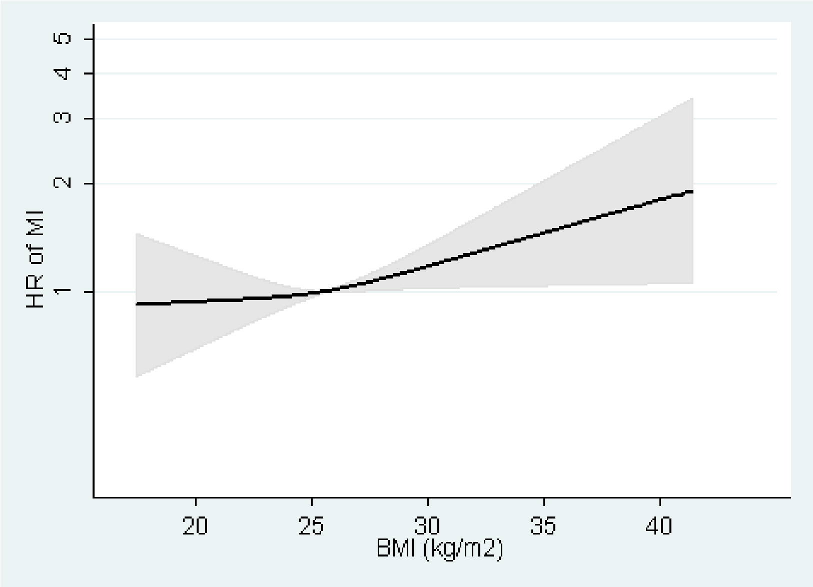

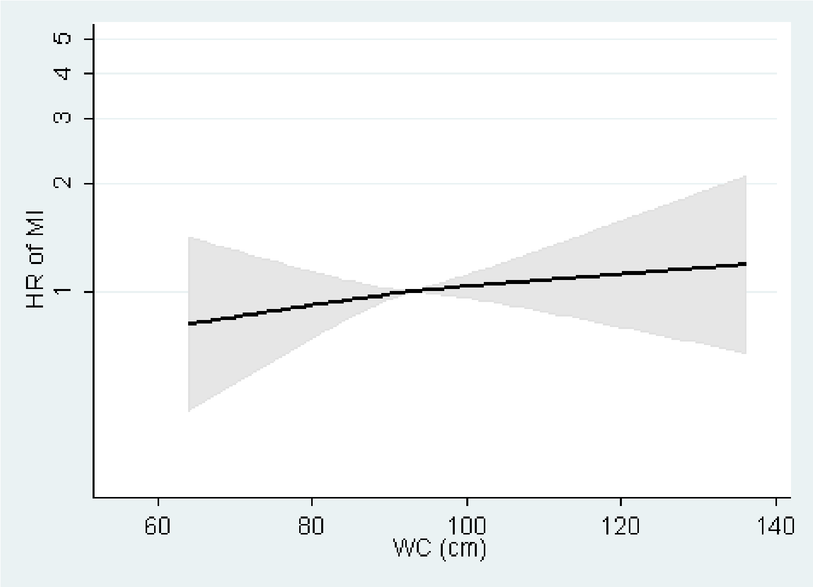

Supplement: Figure S3 — Hazard ratios (HR) and 95% confidence intervals (CI) of myocardial infarction (MI) according to body mass index (BMI) and waist circumference WC) in 1999–02 with mutual adjustment. Abbreviations: BMI, body mass index. HR, hazard ratio. MI, myocardial infarction. WC, waist circumference. Lines are the hazard ratios (shaded areas the 95%-confidence intervals) derived from Cox proportional hazard models with BMI and WC included as restricted cubic splines (3 knots). Reference points are the means of BMI and WC. Adjusted for: sex, years between examination, age, chronic diseases, smoking, WC (only BMI) and BMI (only WC). (PDF) [file pone.0026849.s003.pdf]
