# Supplementary material for: Changes in Waist Circumference and the Incidence of Acute Myocardial Infarction in Middle-Aged Men and Women
Source: PLoS One. 2011 Oct 26;6(10):e26849. doi: 10.1371/journal.pone.0026849 (PMC3202570; doi:10.1371/journal.pone.0026849)
Supplement: Table S1 — Hazard ratios (HR) and 95% confidence intervals (CI) of myocardial infarction according to changes in waist circumference (DWC) when cases occurring in the first one to five years of follow-up are excluded. Abbreviations: CI, confidence interval. DWC, changes in waist circumference. HR, hazard ratio. * Adjusted for sex, years between examinations, age, chronic diseases, body mass index and waist circumference in 1993–97, changes in body mass index, smoking, Mediterranean diet score, energy intake, education, drinking pattern, sports activity. (PDF) [file pone.0026849.s004.pdf]

Table S1. Hazard ratios (HR) and 95% confidence intervals (CI) of myocardial infarction according to changes in waist circumference (DWC) when cases occurring in the first one to five years of follow-up are excluded

**Years excluded (cases)**	**1y (n=934)**<br>**HR (95%CI)***	**3y (n=683)**<br>**HR (95%CI)***	**5y (n=226)**<br>**HR (95%CI)***
DWC (5 cm)	1.02 (0.95, 1.09)	1.01 (0.94, 1.09)	1.01 (0.92, 1.11)

Legend table S1

Abbreviations: CI, confidence interval. DWC, changes in waist circumference. HR, hazard ratio.

* Adjusted for sex, years between examinations, age, chronic diseases, body mass index and waist circumference in 1993-97, changes in body mass index, smoking, Mediterranean diet score, energy intake, education, drinking pattern, sports activity.
